# Heterogeneous Response to a Quorum-Sensing Signal in the Luminescence of Individual *Vibrio fischeri*

**DOI:** 10.1371/journal.pone.0015473

**Published:** 2010-11-16

**Authors:** Pablo Delfino Pérez, Stephen J. Hagen

**Affiliations:** Physics Department, University of Florida, Gainesville, Florida, United States of America; Yale School of Medicine, United States of America

## Abstract

The marine bacterium *Vibrio fischeri* regulates its bioluminescence through a quorum sensing mechanism: the bacterium releases diffusible small molecules (autoinducers) that accumulate in the environment as the population density increases. This accumulation of autoinducer (*AI*) eventually activates transcriptional regulators for bioluminescence as well as host colonization behaviors. Although *V.fischeri* quorum sensing has been extensively characterized in bulk populations, far less is known about how it performs at the level of the individual cell, where biochemical noise is likely to limit the precision of luminescence regulation. We have measured the time-dependence and *AI*-dependence of light production by individual *V.fischeri* cells that are immobilized in a perfusion chamber and supplied with a defined concentration of exogenous *AI*. We use low-light level microscopy to record and quantify the photon emission from the cells over periods of several hours as they respond to the introduction of *AI*. We observe an extremely heterogeneous response to the *AI* signal. Individual cells differ widely in the onset time for their luminescence and in their resulting brightness, even in the presence of high *AI* concentrations that saturate the light output from a bulk population. The observed heterogeneity shows that although a given concentration of quorum signal may determine the average light output from a population of cells, it provides far weaker control over the luminescence output of each individual cell.

## Introduction

Numerous bacterial species use a form of chemical communication known as quorum sensing (*QS*) to regulate gene expression [1]. The bacteria synthesize and release small diffusible molecules known as autoinducers, which accumulate as the bacterial population density grows. As their concentration rises, the autoinducers activate transcriptional regulators that trigger important phenotypic changes in the cells. *QS* therefore allows a population-sensitive switch between different phenotypic states [1]. However, although *QS* is most easily interpreted as a population-counting behavior, *QS* pathways are typically complex, often employing multiple autoinducer signals and receptors. They may also interact with other physical and biological parameters of the organism's environment in addition to the population density [2]–[5].

The complexity of these pathways raises questions about how bacteria use *QS* to probe their environment and exactly what types of information they may gather through this mechanism. Understanding the capabilities and fundamental limitations of *QS* requires detailed experimental and theoretical studies of *QS* systems at the level of individual cells. The goal of this study is to characterize the overall performance of *QS* at the single-cell level in one important model organism. We aim to measure the precision with which an individual *Vibrio fischeri* cell converts a well-defined *QS* signal input to a bioluminescence output.

*V.fischeri* is a Gram-negative marine bacterium that regulates its own bioluminescence through *QS* [6]. The luminescence is produced by a bacterial luciferase that utilizes FMNH_2_, O_2_, and a long-chain aldehyde as substrates. At low cell densities, as in open seawater, the *lux* genes that synthesize the luciferase and substrates are switched off and the bacterial cells are dark. However, the bacterium also colonizes the light organs of fish and squid species, where it attains high cell densities and the *lux* genes become strongly induced. In the light organ of its symbiotic host squid *Euprymna scolopes, V.fischeri* may attain 10^9^–10^10^ cells/cm^3^ and a single cell may emit ∼10^3^ photons/s [7].

**Figure 1:**
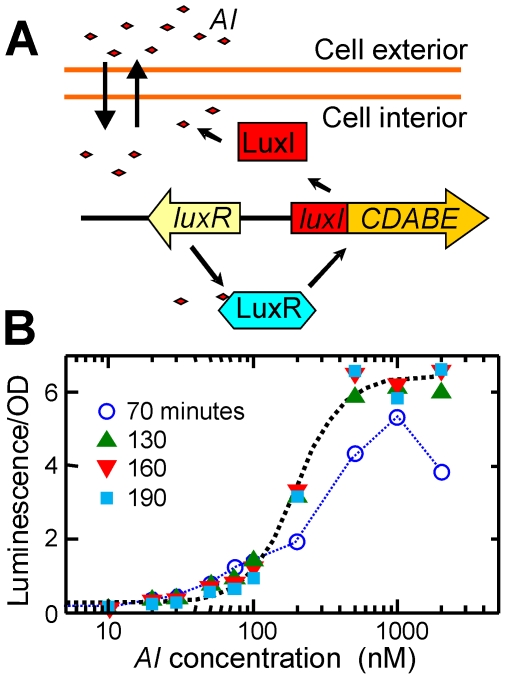
Schematic of LuxI/LuxR regulation of *V.fischeri* bioluminescence, and bulk response. (*A*) LuxI synthesizes the autoinducer *AI* (*N*-3-oxohexanoyl-*L*-homoserine lactone) which binds to LuxR, the transcriptional activator for the luminescence genes *luxCDABE* [6]; (*B*) Luminescence response of a bulk culture of *V.fischeri* strain MJ11 growing in defined medium at room temperature. The points show the response of a (bulk) population of exponential phase cells in a 48-well plate, following addition of exogenous autoinducer (*AI*) at time *t* = 0. After 70 minutes an *AI*-dependent response is developing. After 130 minutes the response has reached a steady state. Data for *t*≥130 minutes are fit to a cooperative binding model (black dotted curve) to give an equilibrium constant *K_eq_* = 200±10 nM and Hill coefficient *n* = 2.6±0.4. Luminescence data are normalized to the optical density at 600 nm to give the luminescence per cell, in arbitrary units.

Studies of bulk populations of *V.fischeri* have revealed an intricate molecular mechanism for this population-sensitive switch [6], [8]. The *QS* pathway employs three autoinducer synthases, three corresponding autoinducers, and three cognate receptors [8]. The full pathway integrates the separate autoinducer signals to regulate not only the luminescence behavior but also other phenotypes related to colonization of the symbiotic host [9]. Of the three signal channels, the LuxI/LuxR pathway shown in **Figure 1A** has been the subject of the most extensive study. It consists of an autoinducer synthase LuxI, an autoinducer (*N*-3-oxohexanoyl-*L*-homoserine lactone, 3OC6HSL), and the transcriptional activator LuxR, as well as the luminescence genes *luxCDABEG*. When the concentration of 3OC6HSL is sufficiently high, it forms a complex with LuxR that activates transcription of the *lux* operon, leading to luciferase synthesis and bioluminescence. The other two *QS* pathways (not shown in **Figure 1A**) detect a second homoserine lactone autoinducer (*N*-octanoyl-*L-*homoserine lactone, C8HSL) that is produced by a synthase AinS and a third autoinducer *AI-2* (as in *V. harveyi* [8]) that is produced by LuxS.

Because it was the first known example of a Gram-negative *QS* system and remains one of the best understood, LuxI/LuxR has been a model system for theoretical and computational studies of the dynamics of quorum regulation. Several authors have modelled its deterministic dynamics [10]–[14] as well as the stochasticity [15]–[17] arising from the biochemical noise in gene expression [18]. The deterministic models characterize the stability of the “on” and “off” states of LuxI/LuxR luminescence as well as the dynamics of switching and hysteresis. Experiments on bulk cultures can provide a suitable test of such models [14]. However, bulk studies measure only average properties of the population. They do not address stochasticity and they do not reveal exactly what information the individual cell gathers in probing its environment with a *QS* mechanism. In particular, the accuracy of the *QS* pathway as a sensor of the individual cell's environment and as a regulator of phenotype, and the impact of stochasticity on *QS*, can only be tested by experimental measurements on individual cells [14], [19]–[21]. Here we ask how accurately the autoinducer signal input to a single cell defines or predicts the bioluminescence output from that cell.

A single-cell study of *V.fischeri* presents technical challenges, as the bioluminescence emission from individual bacterial cells is exceedingly weak and has rarely been measured quantitatively [22]–[25]. The light output from one *V. fischeri* cell is estimated to lie in the range from 10^−2^ to 10^4^ photons/s, depending on the strain, the environment, and whether the culture is fully induced by its multi-input *QS* system [7], [26]. Only a fraction of this photon flux can actually be collected, and therefore the measurable flux from one cell is typically weaker than the signal that can be collected from even a single molecule of a fluorescent reporter like EGFP [27], [28]. Under stable conditions and with sufficiently long integration times, however, the luminescence from one cell can be measured with a photomultiplier [24] or with an intensified or cryogenically-cooled CCD camera [22], [23], [29]. We used an intensified camera and long image exposures (10–15 minutes) to track the bioluminescent emission from individual cells of *V.fischeri* strain MJ11. The cells were immobilized on the window of an observation chamber that was continuously perfused with medium containing exogenous 3OC6HSL autoinducer (*AI*), so that each cell was subject to a precisely defined local *AI* concentration. Tracking individual cells over periods of several hours, we found that cells differ widely in the time scale of their bioluminescence response and in the overall intensity of that response. Hence, while *QS* can coordinate and synchronize the average luminescence output of the bacterial population, it has relatively imprecise control over the response of an individual cell.

## Results

Individual bacterial cells emit very weak bioluminescence and the corresponding signal levels are far weaker than (*e.g.*) the fluorescence that is typically collected from a cell expressing GFP. Therefore, as explained in the *Materials and Methods* and Text S1, we used several procedures to ensure that the microscopy imaging and alignment were stable over the 3–4 hr period of luminescence observations and that any observed heterogeneity in the light output from individual cells was not a detection artifact. We verified that the cells remained stationary and in focus during imaging (**Figure S1**), that the observed variations in luminescent emission were larger than our measurement uncertainties (**Figure S2**), and that the camera, microscope, and images were physically stable over periods of 4 hours or longer.

**Figure 2:**
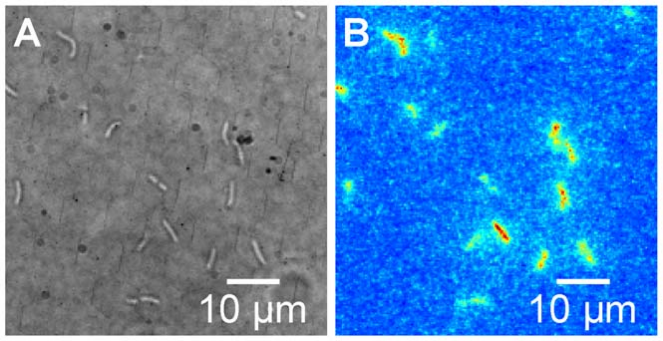
Individual *V.fischeri* imaged in dark field and bioluminescence. (*A*) Dark field (externally illuminated) and *(B*) bioluminescence (light emission) images of *V.fischeri* cells adhered to the glass window of the perfusion chamber at 24°C in the presence of 500 nM exogenous *AI*. The cells appear as rods (∼3–5 µm long) in the dark field image. The bioluminescence image shows in false color the luminescent emission detected in a 16 minute exposure. Images were collected in an inverted microscopy configuration with an intensified CCD camera and a 100× oil immersion objective.

In the absence of exogenous autoinducer the *V.fischeri* cells in the perfusion chamber produced no detectable luminescence. However, when at least ∼50 nM of autoinducer (*AI*, 3OC6HSL) was provided in the flowing medium the luminescence of individual cells was clearly resolved. **Figure 2** compares dark-field (*i.e.* externally illuminated) and bioluminescence (*i.e.* luminescence emission without external illumination) images of individual *V. fischeri* cells adhering to the glass window in the presence of 500 nM *AI*. Qualitatively the image already suggests that different cells emit with different intensities, even at a high *AI* concentration that saturates the output of the bulk population (**Figure 1B**). A quantitative analysis of all data confirmed that the brightness of the cells was heterogeneous at all autoinducer concentrations studied (0–1000 nM *AI*). At 1000 nM *AI* we found many individual cells emitting little light during a ten minute exposure, even though we observed these same cells growing and dividing during the ∼4 hr duration of observation.

**Figure 3:**
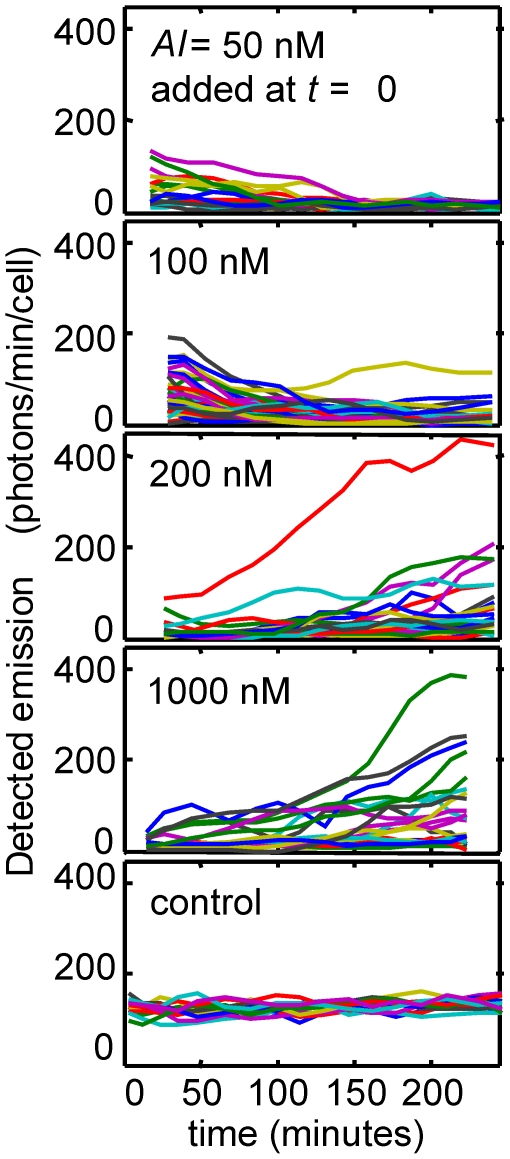
Luminescence of individual *V.fischeri* cells following addition of autoinducer, and detection stability test. At each autoinducer concentration, roughly 25–40 MJ11 cells were imaged repeatedly over a period of ∼4 hrs following introduction (at *t* = 0) of exogenous autoinducer *AI* at the indicated concentration. The light emission from each cell was quantified through analysis of a series of 10-minute camera exposures (see *Materials & Methods*). The state of induction of the initial cell culture determines the luminescence of the cells at *t* = 0. However, once adhered in the flow chamber and exposed to the flow of medium (containing exogenous *AI*), the cells respond by adjusting their luminescent output. This leads to a transient increase or decrease in the emission over the next ∼1–2 hrs. After ∼3 hrs the cells have adapted to the applied *AI* level. The control shows an experimental verification of the stability and sensitivity of microscopy and data analysis. For this measurement, green fluorescent latex spheres were illuminated with a severely attenuated blue light source and then imaged with the same camera settings, magnification, 10-minute exposure time, and data analysis, as used for the *V.fischeri* measurements. Image focus and excitation intensity were not adjusted during the 4 hr measurement. Twelve representative trajectories are shown. See *Materials and Methods* and Text S1. The time-dependence of all emission *versus* time “trajectories” in this figure has been smoothed by a Gaussian filter with width σ = 10 minutes.

Studies of bulk cultures under our growth conditions established that the shape of the luminescence *versus AI* response curve was established within 2–3 hrs following introduction of *AI* (**Figure 1B**). Therefore, an observation period of ∼3–4 hrs in a perfusion chamber should be sufficient to observe the response of individual cells to introduction of *AI*. **Figure 3** shows the time course of the luminescence collected from an ensemble of individual cells. The luminescence of each cell is tracked over time through a series of 10-minute camera exposures (see *Materials and Methods*), following the introduction of exogenous *AI* at *t* = 0. The initial response of the cells (0≤*t*≤100–150 minutes) is a transient increase or decrease in average luminescence, as the *AI* concentration in the perfusion chamber may be greater or less than in the starting culture. On a longer time scale (*t* = 150–250 minutes) the cells attain average emission levels that are consistent with the supplied concentration of exogenous *AI*. However a large degree of cell-to-cell variability is apparent. The brightness of the different cells diverges over time, with many cells luminescing at very modest levels while a small fraction of cells emit much more brightly.

**Figure 4:**
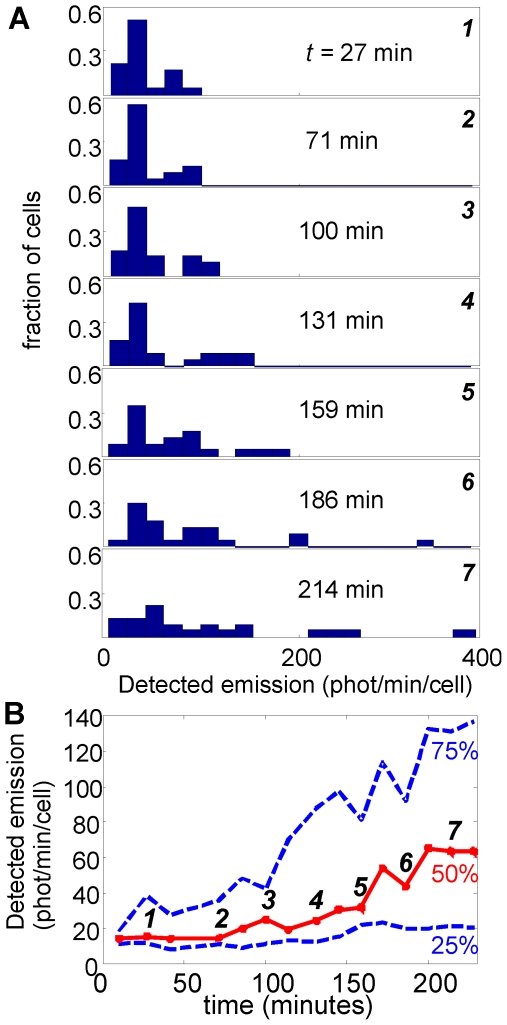
Spreading of the luminescence histogram over time. (*A*) Cell brightness histograms for MJ11 cells at the indicated times, following introduction of 1000 nM *AI* at *t* = 0. (*B*) Median (red curve) cell brightness and the 25% and 75% percentiles of brightness (blue curves). The distribution of intensities broadens as the cells response to the exogenous *AI* signal. A substantial fraction of the cells emit near the detection threshold (∼10–20 photons/minute/cell) even at *t* = 4 hrs.

**Figure 5:**
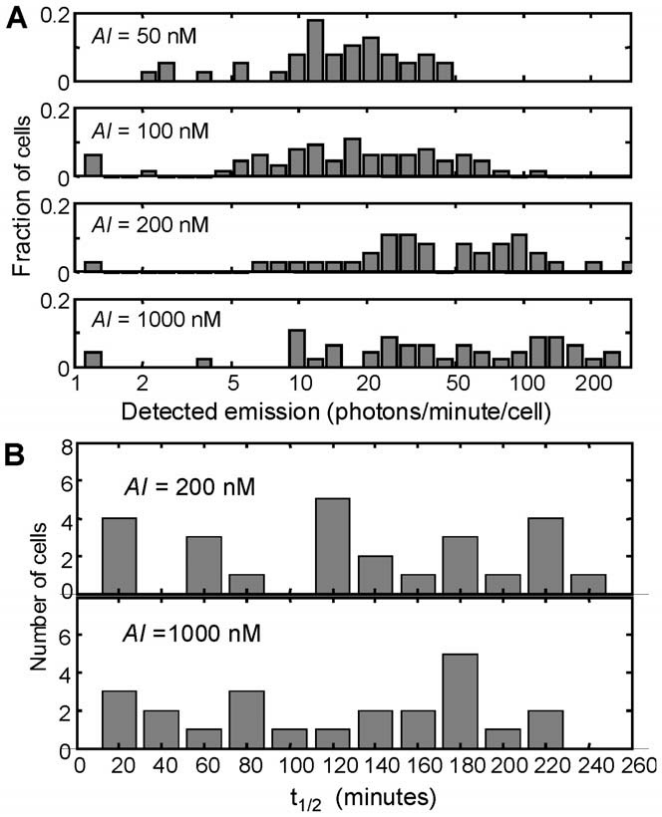
Histograms of luminescence levels and onset times at high autoinducer concentration. (*A*) Distribution of luminescence levels detected for individual *V.fischeri* cells, at time *t* = 240 minutes after autoinducer (*AI,* 3OC6HSL) was introduced at concentrations indicated. Cells emitting ∼10–20 photons/minute are at the measurement uncertainty, *i.e.* are consistent with no emission. (*B*) Distribution of luminescence onset times *t*_1/2_ in the presence of 200 nM and 1000 nM *AI.* The onset time *t*_1/2_ is the time at which the luminescence output *I*(*t*) of a particular cell is halfway between its initial value *I*(*t* = 0) and its final value *I*(*t*≈250 minutes), when *AI* was introduced at *t* = 0.

Heterogeneity is also apparent in the time scale of response. **Figure 3** shows that, when a high *AI* concentration (1000 nM) is introduced at *t* = 0, some cells begin to respond quickly, with 250–350 photons/minute detected after 250 minutes. Other cells however are only beginning to respond after ∼150 min. **Figure 4** shows the progression of the brightness distribution as a group of cells responds to the introduction of 1000 nM *AI.* The variability in the time scale of response (the kinetic heterogeneity) can be summarized by the distribution of the onset time *t_1/2_*, which we define as the time at which the luminescence of a particular cell is halfway between its initial (*t* = 0) and final (*t*≈250 minutes) values. **Figure 5B** shows that *t_1/2_* has a very broad and flat distribution at 200 nM, and this distribution remains broad even at a saturating *AI* concentration of 1000 nM.

Hence we observe several types of heterogeneity in the response of *V.fischeri* to a defined *AI* concentration. Cells in the same environment respond on widely differing time scales when *AI* is introduced, and they also differ in the overall amplitude of that response. Furthermore the individual trajectories of **Figure 3** suggest that the luminescence of at least some cells occasionally fluctuates by ∼20–40% on time scales of ∼30 minutes.

As shown in Text S1 and **Figure S3**, our experimental configuration also allows us to observe other kinetic and steady state phenomena in single-cell *V.fischeri* luminescence, such as the “rich medium effect” [30]–[32]. However we focus here on the heterogeneity of the *QS* response.

## Discussion

The luminescence of *V.fischeri* is activated through a quorum sensing (*QS*) mechanism in which the cells remain dark until their population reaches the high densities that signify colonization of the light organ of the symbiotic host. Here we ask how tightly this *QS* system regulates the luminescence output of an individual cell in response to a defined chemical signal (*i.e.* the 3OC6HSL autoinducer concentration). We find that an ensemble of cells produces a distinctly heterogeneous response to the *AI* input, with significant cell-to-cell variability in the overall level of emission and in the onset time for this response, as well as indications of short term fluctuations in brightness.

In the absence of exogenous *AI* the light emission from the cells was below measureable levels. However, after ∼150–250 minutes in exogenous *AI* the individual cells were significantly brighter on average, as in a bulk culture. The addition of *AI* not only increases the average brightness, but also increases the (absolute) differences in the brightness of individual cells; hence the individual brightness levels eventually span an order of magnitude, as shown in **Figure 5A**. Similarly the luminescence onset time *t*_1/2_ shows a broad distribution at both 200 nM and 1000 nM *AI* (where the response of the bulk population in **Figure 1B** is seen to saturate). As the distributions for both the individual cell intensities and the onset times in **Figure 5B** are not at all clustered about the mean values they are clearly not Gaussian (normal) distributions.

**Figure 6:**
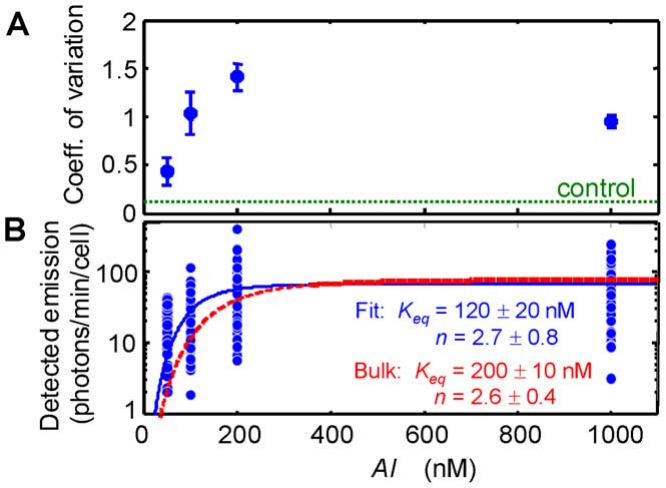
Variation and mean of luminescence levels *versus* autoinducer concentration. (*A*) Coefficient of variation (*cv* = standard deviation/mean) in the luminescence of different cells. Variation is calculated from emission levels recorded *t >*100 minutes after introduction of exogenous *AI;* (*B*) Luminescence emission detected from 188 individual cells (blue circles) after 150–250 minutes exposure to *AI*. Data for each *AI* concentration represents a different group of cells. Solid curve (blue) is a fit to a cooperative binding model, giving *K_eq_*≈120±20 nM and Hill coefficient *n*≈2.7±0.8. For comparison with the expected average behavior, the dashed curve (red) shows the *AI* response that is obtained from a bulk population after 150–250 minutes in autoinducer (**Figure 1**).

Nevertheless these single-cell data are still consistent with the behavior of a bulk culture, as can be seen by comparing the *AI* response curves of single cells and a bulk culture under the same growth conditions. **Figure 6B** shows that a nonlinear least squares fit of a cooperative binding model to the single-cell data gives an equilibrium constant *K_eq_*≈120±20 nM and a Hill coefficient *n*≈2.7±0.8. By comparison, the average luminescence of a bulk culture of the same strain (**Figure 1B**) gives *K_eq_*≈200±10 nM and *n*≈2.6±0.4. The smooth *AI*-induced luminescence response of the bulk population is a result of averaging over large numbers of cells; it conceals a very heterogeneous character in the response of individual cells in that population.

We find it remarkable that such large variations in emission persist in a homogeneous *AI* environment, even several hours after introduction of the exogenous signal. Even though we anticipate that stochasticity will generate cell-to-cell variability, the coefficient of variation (*cv* = standard deviation/mean ∼1) in **Figure 6A** appears much greater than is expected from stochastic simulations of the LuxI/LuxR system. For example, Cox *et al.* estimated the kinetic parameters for a chemical model of the LuxI/LuxR network [15]. Their stochastic simulations predicted relatively modest variability in the activation of *luxI* as a function of *AI* concentration. Although the LuxI concentration was variable at low (<50 nM) *AI* concentrations, the simulations predicted minimal fluctuation, with a standard deviation less than ∼10% of the LuxI concentration once the *AI* concentration reached the induction threshold. By contrast we find a large variation in light output persisting across the *AI* induction curve. The *cv* of the luminescence is near unity even at 1000 nM *AI.* This variability is presumably not attributable to heterogeneity in intracellular *AI* concentrations, as the *AI* diffuses rapidly across the cell membrane [32] and the exogenous *AI* level is well-controlled by the flow of medium.

Our emission *versus* time trajectories also show some evidence for short-term fluctuations in the single-cell luminescence. The time series data of **Figure 3** suggest that the light output from some cells occasionally fluctuates by ∼20–40%. Furthermore, while the brightness of each cell is reasonably stable on short time scales, the brightness of one cell is poorly correlated with its brightness ∼30–60 minutes later (Text S1 and **Figure S4**). An early study of the time dependence of *V. fischeri* luminescence found no significant oscillation or pulsing in the luminescence output at frequencies 0.01–10 Hz, although it did not investigate the low frequency behavior (∼10^−3^ Hz) studied here [24]. Whether the noise in *lux* gene expression does in fact have a bursting or intermittent character under stable environmental conditions is an intriguing question that requires further study. However the short time scale of these fluctuations suggests that they originate in intrinsic (*i.e.* purely biochemical stochastic) noise [33]. By contrast the slower intercellular variability in the onset times for *AI* response and in the overall luminescence output is more suggestive of extrinsic noise originating in the variable concentrations of cellular components such as ribosomes, polymerases, or in different stages in the growth cycle, etc. [34].

A recent study of the *QS* bioluminescent emission of individual *V. harveyi* also found very substantial cell-to-cell variability [19]. Anetzberger *et al.* allowed *V. harveyi* cells to accumulate their own autoinducer for intervals up to 8 hours and reach quorum conditions. This produced an approximately bimodal response, with many cells luminescing brightly while roughly 25% of live cells remained relatively dark, or roughly one-tenth as bright as the more luminescent cells. Although the LuxI/LuxR pathway probed here has a different structure from the *lux* regulatory system of *V. harveyi* (*i.e.* LuxI/LuxR does not directly include the type of phosphorelay switch and sRNA regulation found in *V.harveyi*), these findings are similar to ours: after several hours in *AI,* roughly 25% of *V. fischeri* cells were emitting luminescence at or below our detection limits (**Figure 4B**). Our results show that this heterogeneity occurs across a range of *AI* concentrations and also extends to the kinetics of the onset of luminescence.

However another recent single-cell study of the *V. harveyi QS* pathway found a more homogeneous response to autoinducer [20]. Long *et al.* constructed a *qrr4-gfp* transcriptional fusion that allowed them to use GFP fluorescence – rather than the native luminescence – to monitor the effect of two autoinducer signals on the activation of the quorum-regulatory RNAs that are controlled by the phosphorylation of LuxO. LuxO phosphorylation is in turn regulated by the three autoinducer receptors in *V.harveyi.* Long *et al.* found much less variance in the response of different cells at the same autoinducer concentrations than Anetzberger *et al.* observed in the bioluminescence response, and much less than we report here in *V.fischeri* luminescence. For the two different *AI* receptor mutants (each responsive to a single autoinducer) that they studied, they observed a coefficient of variation *cv* ∼0.2–0.4 in the *gfp* expression, significantly smaller than the *cv* ∼1 that we observe here in *V.fischeri* luminescence.

To explain the observation of heterogeneity in the luminescence (but not in the *gfp* reporter strains) of *V.harveyi*, Anetzberger *et al.* suggested a possible role for positive feedback in the *V.harveyi* master regulator LuxR (not homologous to *V.fischeri* LuxR), which is regulated by the sRNAs and controls expression of the *lux* genes for luminescence. They proposed that the absence of autoinducer synthases in the GFP reporter strains eliminated possible feedback loops involving *AI* synthesis and detection, leading to a more homogeneous behavior in those strains. The fact that our system defines the *AI* concentration exogenously – also eliminating *AI* feedback – yet still exhibits heterogeneity argues against this interpretation. However a role for feedback in the observed noise is nevertheless plausible in LuxI/LuxR. Williams *et al.* recently studied the dynamics of *AI* sensing by an *E.coli* model strain *lux01*, in which LuxR is activated by 3OC6HSL to control the expression of *gfp* while the autoinducer synthase LuxI is absent [14]. Cell cytometry studies found a bimodal response of *gfp* expression to the *AI* signal level, with the more responsive cells exhibiting a roughly log-normal distribution in GFP fluorescence. They argue that the external *AI* concentrations feed into an autoregulatory feedback loop for LuxR expression, and that this generates hysteresis in the LuxI/LuxR system's response to *AI*. That is, its activation at any particular *AI* concentration depends in part on its prior history and initial LuxR levels. This LuxR mechanism would help to explain some of the cell-to-cell variability that is observed in the luminescence onset time in **Figure 5B**, as natural stochastic variations in initial LuxR levels would be amplified by feedback to give large changes in activation of the luminescence genes.

**Figure 7:**
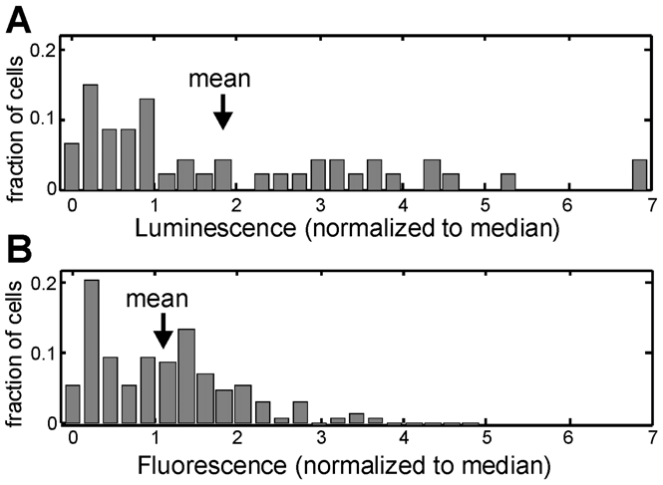
Heterogeneity of native luminescence *versus* fluorescence reporter for *V.fischeri* quorum system. (A) Histogram of bioluminescence emission levels from 47 individual *V.fischeri* cells of wildtype strain MJ11, following induction by 1000 nM *AI.* The luminescence levels are normalized to the median value. (B) Histogram of fluorescence levels for 127 individual *V.fischeri* cells of mutant JB10, following induction by 1000 nM *AI.* The JB10 mutant contains a chromosomal *gfp* insertion between *luxI* and *luxC* in the LuxI/LuxR system. Fluorescence values are normalized to the median value. Both luminescence and fluorescence reporters for the *QS* system show a broadly heterogeneous response at full induction, although the fluorescence shows slightly less variability (*cv*≈0.8) than the luminescence (*cv*≈1.0).

Alternatively it is possible that the heterogeneity in light output results from some differences in the energy resources of different cells, with some cells in bright (energy-intensive) states and others in dark (recovering) states. However our data suggest that the overall luminosity state (brighter or darker) of a cell tends to persist over relatively long periods of hours, comparable to the doubling time. Cycles of energy depletion and recovery would presumably play out over shorter time scales. We also found that the output variability was not due to a shortage of the C14 long chain substrate needed for the luciferase reaction (see Text S1). Furthermore, heterogeneity was not exclusive to a luminescence reporter of the LuxI/LuxR system: under full induction of LuxI/LuxR, the expression of a *gfp* reporter by *V.fischeri* mutant JB10 showed heterogeneity (*cv* = 0.8) similar to that of the bioluminescence (**Figure 7**). These points suggest that cell-to-cell variability in luminescence response is not primarily due to a deficiency of the luminescence substrate or energy resources.

Our findings raise some interesting questions about the performance of *V.fischeri QS* at the single cell level. For example, the broad heterogeneity in the light output from the cells – which always remained short of the estimated maximum output of ∼1000 photons/s/cell [7] – raises the question of whether the observed heterogeneity is still present in cultures emitting at maximum brightness (*e.g.* within the symbiotic host). It would also be interesting to determine whether the two other signal inputs in *V. fischeri*, *i.e.* the C8HSL and *AI-2* autoinducers, drive a similarly noisy response or whether they improve the noise performance of the overall system. Mehta *et al.* recently argued that the processing of information by the *QS* system of *V. harveyi* is limited primarily by interference between the three input signal channels of the *QS* pathway, and secondarily by noise originating within each pathway [35]. Because noise in any one signal input channel ultimately feeds forward into the regulated output, a well-defined input concentration for one of the three autoinducer species will not ensure a predictable output. In the present *V. fischeri* study we have defined the 3OC6HSL level externally and also set the other two autoinducer concentrations (C8HSL and *AI-*2) virtually to zero by advection; hence it appears unlikely that these additional receptors contribute significant noise to the luminescence output.

The heterogeneity observed here may also argue against an interpretation of the LuxI/LuxR system – or at least its regulation of the bioluminescence genes – as allowing an individual cell to acquire much useful information about its local microenvironment [4], [5], [36]. The individual luminescence response seems to contain little information about (*i.e.* it is a poor indicator of) the local *AI* level, just as the *AI* concentration is a weak predictor of the luminescence response. If a group of cells in a well-defined and homogeneous environment exhibit widely divergent responses, one cannot consider the *QS* system to be a reliable sensor of local diffusion constants, for example. In a more heterogeneous natural microenvironment one expects that the cell-to-cell variability in response would only increase.

There are scenarios in which phenotypic variations arising from noisy gene expression can provide a tangible benefit to the cell [37], [38]. Therefore it is intriguing to consider whether noise in *V.fischeri* luminescence benefits the bacterium or influences its symbiosis with a host animal. In the symbiotic relationship *V.fischeri* is subject to a strong selective pressure to maintain bright luminescence. For example, the squid *E.scolopes* does not tolerate colonization by dark mutants of *V.fischeri* [39], [40]. However, although the host can select a strain for its average luminescence output, the squid presumably cannot detect temporal or other types of heterogeneity at the single-cell level. It may detect the mean – but not the variance – of the cell brightness. Therefore the individual cell is not likely to endure host pressure to minimize its brightness fluctuations. Thus one possible interpretation of our results is that the signal response is poorly coordinated across the population because the host cannot apply feedback to enforce tight coordination.

Of course, this interpretation only raises the question of whether the uncoordinated response brings any benefit to the bacteria. It would be interesting to determine whether cells that emit a weak luminescence are directing more energy into other *QS*-regulated behaviors, as if to divide colonization tasks across the population. Alternatively, since bright emission is energy intensive, one may speculate that a form of *QS* cheating occurs, with the less luminous cells enjoying a growth advantage. In a fully induced cell the luminescence may require more than ∼10^4^ ATP molecules per second and account for up to ∼20% of the oxygen consumption [7]. Such cheating does appear to provide a benefit to individual bacteria, although it is expected to be less pervasive in clonal populations where kin selection favors cooperation [41]. (Cultures grown from a single colony of MJ11 were as heterogeneous in light output as cultures grown from multiple colonies, as described in Text S1.) Finally, a variable luminescence output could be an optimal strategy in fluctuating environments or when some of the autoinducer signals are weak or absent, so that the cell's obligation to luminesce is uncertain. Noisy output would be less advantageous in the rich, supportive environment of the host light organ.

In summary we have observed that the luminescence response of individual, wild-type *V.fischeri* cells is very imprecisely regulated by the local quorum signal level. As *QS* regulation plays an important role not just in the bioluminescence of *V. fischeri* but also in colonization of the symbiotic host [9] it will be interesting to conduct mutational studies to investigate whether the noisy behavior observed in this particular output also extends to other targets of *QS* regulation in this organism, and how this influences the organism's ability to colonize the heterogeneous microscopic environment within the host light organ.

## Materials and Methods

*Vibrio fischeri* strain MJ11 (NCBI Taxonomy ID: 388396), a strain derived from the host fish *Monocentris japonicus* [42], was provided by Prof. M. Mandel and Prof. E. Ruby. Cells were prepared in exponential phase at 24°C in defined artificial seawater medium [43] containing glycerol as carbon source. Approximately 15 µl of culture in exponential phase was deposited at the center of the lower window of a perfusion chamber. This chamber consisted of a cylinder (volume approximately 1.5 cm^3^) constructed from two parallel, circular coverslips (25 mm diameter) spaced 5 mm apart. The lower window was coated with poly-*L*-lysine to promote adherence of the cells. The chamber was then closed and the cells were allowed to settle and adhere to the window. After ∼15 minutes the chamber was then washed with approximately two chamber volumes of defined medium from a programmable syringe pump. This wash diluted away any autoinducer that was present in the starting culture and removed any non-adhering cells. The chamber was then placed on the stage of an inverted microscope and the pump flow rate was reduced to 0.2 ml/hr in order not to disturb the adhered cells during observation. The cells in the chamber were primarily located within a small area (few mm^2^) of the window, directly above the microscope objective, which was an infinite-conjugate 100× plan oil immersion objective, NA 1.25. The blue/green (near 490 nm) bioluminescence from the cells on the lower window was collected by the objective and focused onto an intensified CCD camera (512×512 pixel, I-MAX-512-T operating at −35°C, Princeton Instruments, Princeton NJ) via an achromatic doublet lens, to give a final image scale of 0.278 µm per pixel.

The concentration of 3OC6HSL autoinducer was selected by adding exogenous autoinducer (*AI, N-(*3-Oxohexanoyl)-*L*-homoserine lactone, CAS 143537-62-6, No. K3007 from Sigma Aldrich, St. Louis) to the medium flowing in the chamber. The continuous flow of medium removed unattached (freely swimming) cells from the chamber and maintained the *AI* concentration at the selected level. *AI* released by the few cells adhered on the glass was efficiently removed by diffusion into the passing flow. This was verified in two ways. First, numerical integration of the diffusion/advection equation for our experimental configuration gives an *AI* accumulation of less than 50 pM at the window (for *AI* synthesis at 10^−21^ g/s/cell and diffusion at 100 µm^2^/s [44]). This concentration is insufficient to induce detectable luminescence. Second, when cells were perfused with medium that contained no added autoinducer, we observed that any luminescent emission from the immobilized cells soon diminished to undetectable levels.

The doubling time for the growth of the cells in the chamber was approximately 2–3 hr, operating at 24°C. This growth rate set a practical limit of roughly 4 hrs to our observations of individual cell luminescence in the perfusion chamber. Once the cells on the window had divided more than once or twice, the cells appeared as clusters and it became difficult to distinguish the luminescence of neighboring cells in the camera images. We studied the luminescence of wild type strains only. Preliminary studies of *V.fischeri* strain ATCC 7744 gave results similar to those presented here for strain MJ11. A fluorescence study of *gfp-*reporter strain JB10 is described below.

In most of our studies, the programmable syringe pump supplied a flow of defined medium containing 0–1000 nM added autoinducer to the chamber. During the “rich medium” study (see Text S1) the syringe delivered commercial photobacterium medium (No. 786230, Carolina Biological, Burlington NC) mixed with defined medium and *AI* as indicated. For the tetradecanoic acid study (see Text S1), we prepared a 1 mM stock solution of tetradecanoic acid (myristic acid, M3128 from Sigma Aldrich) in ethanol and diluted this 1000× into the defined medium, to give a final concentration of 1 µM.

After placing the perfusion chamber on the microscope stage and starting the flow of medium + *AI*, we used dark field images (*i.e.* externally illuminated images with brief exposure times) to locate and focus on individual cells. We then disconnected the illumination source and collected a bioluminescence image (*i.e.* collecting only bioluminescent emission) with an exposure time of (typically) ten minutes, and then collected another dark field image for comparison. **Figure 2** and **Figure S1** show sample images. We repeated this process over a period of ∼4 hrs for each group of cells (at a fixed *AI* concentration), collecting alternately both dark field and bioluminescence images at regular intervals. Comparisons of successive dark-field images provided a running check of the physical and optical stability of the cells and the scene being imaged.

To quantify the emission levels of individual cells in the bioluminescence images, we first used the dark field images to obtain the pixel coordinates of individual cells that had remained immobile during the experiment. We then defined a small rectangular region surrounding each cell. We binned (2×2, to improve SNR) the pixels of the corresponding region within the dark-subtracted luminescence image, generated the brightness histogram of the pixels in that region, and fit the lower portion (only) of that histogram to a Gaussian distribution. This distribution accurately models the background intensity distribution in cell-free regions of the image. We then subtracted the fit Gaussian from the actual histogram and summed the residual. This provided a satisfactorily robust count of the luminescence emission of a single cell, typically 10–100 photons/minute/cell. We confirmed that the luminescence emission count from a single cell was insensitive to the precise size of the rectangular image region used to estimate that count. Thermally generated background (*e.g.* dark noise) in the CCD image contributes some uncertainty to this emission count. By applying the above data analysis to several image regions that contained no cells, we estimated the magnitude of this uncertainty as ∼20 photons/minute peak-to-peak per cell per image frame. This defines a baseline noise level, prior to Gaussian filtering of the emission level *versus* time record (“trajectory”) of a cell. The image intensifier itself also contributes some noise, which is best characterized by imaging a stable light source, as discussed below. Camera readout noise and photon shot noise were smaller than either of the above noise sources.

We typically detected ∼10–100 photons/minute/cell from *V.fischeri* strain MJ11 in our flow chamber, even in the presence of an *AI* concentration (1000 nM) that would saturate the output of a bulk culture. Therefore, our single-cell luminescence measurements involved signal levels that were drastically lower than are commonly obtained in gene regulation studies using fluorescent proteins like GFP. For this reason it was important to verify that the detected signals and their variations were not due to experimental or analysis artifacts. Text S1 provides further detail on measures that we took to ensure the stability of the optical configuration, with minimal drift in the focus and minimal movement in the cells adhered to the glass. These included collecting and comparing a series of dark-field images (*i.e.* one externally-illuminated dark-field image between each pair of luminescence images) to check that cells under observation remained in focus and had not physically moved.

Text S1 also describes control experiments to verify the stability and sensitivity of our detection system. That is, we verified that the observed variations in the light output from individual *V.fischeri* cells were representative of cellular emission, and were not generated within the image intensifier or due to uncertainty in our detection or analysis. A suitable control must be a micron-sized light source that is comparable in size to the *V.fischeri* cells, feebly luminescent (no brighter than the weak luminescence of a single *V.fischeri* cell), and absolutely stable in its output. For this purpose we used micron-sized green fluorescent latex spheres (FluoSpheres, Invitrogen Inc.) dispersed at low density onto the lower window of the perfusion chamber and illuminated with a heavily attenuated blue LED excitation source. Under exceedingly faint excitation the fluorescence from these spheres in a ten minute camera exposure was comparable in magnitude to the emission detected from individual *V.fischeri* (*i.e.* ∼100 photons/minute/particle) and it remained stable for extended periods. We imaged these spheres with exactly the same instrumentation parameters (camera gain and temperature, exposure time, magnification, etc.) as used for the *V.fischeri* cells. Performing the same image analysis as used for the live cell images, we obtained a highly stable and consistent photon count from the spheres. **Figure 3** shows that the emission detected from the control spheres remained stable through more than four hours of observation, without any manual adjustment of microscope focus. After Gaussian filtering (width σ = 10 minutes) of all emission *versus* time trajectories, the noise level (standard deviation) for the emission from the individual particles was 10–12 photons/minute. **Figure S2** shows that the emission from different spheres in the same image was closely similar as expected (standard deviation/mean≈0.12). These results show that the microscopy system and the data analysis were sufficiently sensitive and stable for resolving heterogeneity in the luminescent emission from different *V.fischeri* cells.

We also used fluorescence microscopy to measure GFP levels in individual cells of *V.fischeri* strain JB10, which was provided by Prof. E. Stabb. In the JB10 mutant a chromosomal *gfp* reporter is placed under the control of the LuxI/LuxR system by insertion between *luxI* and *luxC*, *i.e. luxI-gfp-luxCDABEG*, so as to express GFP when the LuxI/LuxR system is activated by 3OC6HSL [45]. Cells were grown overnight in the same defined medium used for the luminescence experiments and then transferred to fresh medium containing 1000 nM exogenous *AI.* After incubating the cells with shaking for ∼2 hrs we dispersed the cells on a coverslip and measured the fluorescence of 127 individual cells, using an inverted microscope with a 60× oil immersion objective and a cooled CCD camera (Micromax, Princeton Instruments).

## Supporting Information

Figure S1 **Sequential dark field and luminescence images for one** ***V.fischeri*** **cell.** (*A*) Dark field and (*B*) bioluminescence images of an individual cell adhered to the window of the perfusion chamber, and (*C*) luminescence levels extracted from these images. (The luminescence trajectory has not been Gaussian filtered.) Images were collected at the numbered time points indicated in (*C*). (TIF)

Figure S2 **Variability in signal levels for** ***V.fischeri*** **cells and for reference particles.** Histograms comparing the luminescent emission from individual *V.fischeri* cells (*A*) to the fluorescent emission under weak excitation of a control sample of individual micron-sized latex spheres (*B*). Each histogram shows the number of individual emission measurements falling into the indicated brightness bin, over a ∼30 minute period comprising three 10-minute camera exposures. (*A*) and *(B*) have the same horizontal scales: All images for both cells and fluorospheres were collected in ten minute exposures using identical camera and microscope settings and image analysis. (For the fluorospheres, we used a highly-attenuated blue LED as excitation source and inserted a Schott longpass filter GG485 into the detection path.) The coefficient of variation for the fluorospheres is 0.12, while the coefficient of variation for the *V.fischeri* cells is 1.3 (200 nM *AI*) and 1.0 (1000 nM *AI*). (TIF)

Figure S3 **Inhibition of** ***V.fischeri*** **bioluminescence by complete (“rich”) medium.** Light emission from individual cells in the perfusion chamber was tracked over time as the flowing medium was switched from an initial (100% defined medium) to a final (70% defined medium, 30% complete medium) composition. *AI* concentration remained 1000 nM at all times. Image times represent the starting time of a 16-minute bioluminescence exposure. The histograms, showing the fraction of observed cells emitting at the indicated level, collapse rapidly as complete medium is introduced. (TIF)

Figure S4 **Temporal autocorrelation of individual cell luminescence.** The emission level *I*(*t*) of a cell at time *t* is compared to its emission at a later time *I*(*t*+τ). Data represent individual cell emission levels measured at least 100 minutes after introduction of 1000 nM *AI*: (*A*)–(*D*) For small values of τ, the data are close to the (best fit) line, indicating that a cell's intensity at time *t* is a reasonably good predictor of its intensity at time *t*+τ. However as τ approaches 40–60 minutes, the scatter around the average line increases, indicating that the brightness of the cell at later times (relative to the average or best fit trend) is poorly predicted by its earlier brightness or by the average behavior of the other cells. The vertical distance *d* of each point from the trend line becomes larger at large τ. Panel (*E*) shows σ_d_, (the standard deviation of *d*) as a function of τ. At high *AI* concentrations the standard deviation continues to grow for many minutes, indicating that the brightness of the cells continues to diverge both from its initial value and from the average growth trend. The σ_d_ of the control (fluorescence spheres) is essentially flat as expected, except for a dip near τ = 10 minutes (due to Gaussian filtering of the trajectories). (TIF)

Text S1 (DOC)
