# Supplementary figures and images for: Heterogeneous Response to a Quorum-Sensing Signal in the Luminescence of Individual Vibrio fischeri
Source: PLoS One. 2010 Nov 16;5(11):e15473. doi: 10.1371/journal.pone.0015473 (PMC2982848; doi:10.1371/journal.pone.0015473)

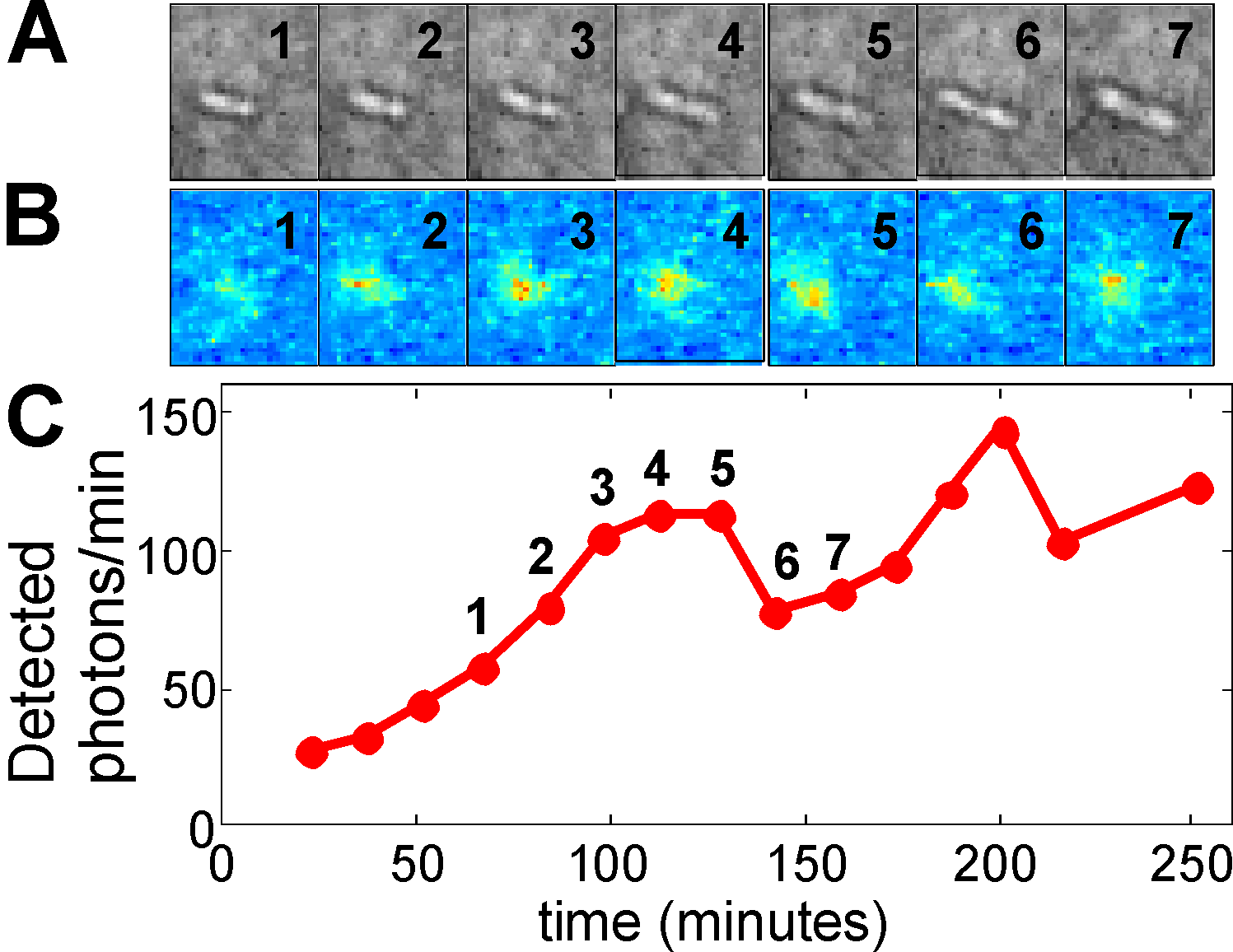

Supplement: Figure S1 — Sequential dark field and luminescence images for one V.fischeri cell. (A) Dark field and (B) bioluminescence images of an individual cell adhered to the window of the perfusion chamber, and (C) luminescence levels extracted from these images. (The luminescence trajectory has not been Gaussian filtered.) Images were collected at the numbered time points indicated in (C). (TIF) [file pone.0015473.s001.tif]

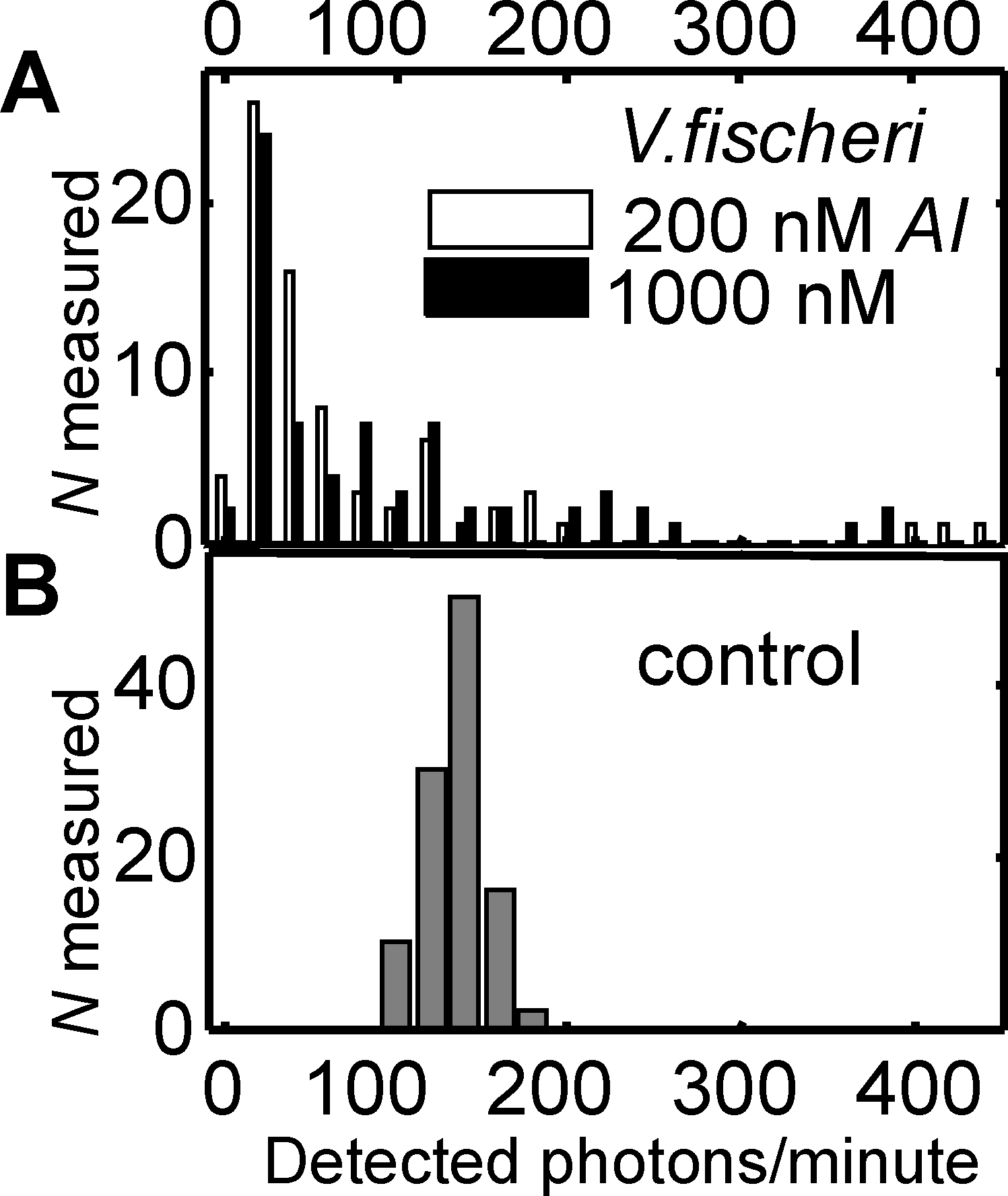

Supplement: Figure S2 — Variability in signal levels for V.fischeri cells and for reference particles. Histograms comparing the luminescent emission from individual V.fischeri cells (A) to the fluorescent emission under weak excitation of a control sample of individual micron-sized latex spheres (B). Each histogram shows the number of individual emission measurements falling into the indicated brightness bin, over a ∼30 minute period comprising three 10-minute camera exposures. (A) and (B) have the same horizontal scales: All images for both cells and fluorospheres were collected in ten minute exposures using identical camera and microscope settings and image analysis. (For the fluorospheres, we used a highly-attenuated blue LED as excitation source and inserted a Schott longpass filter GG485 into the detection path.) The coefficient of variation for the fluorospheres is 0.12, while the coefficient of variation for the V.fischeri cells is 1.3 (200 nM AI) and 1.0 (1000 nM AI). (TIF) [file pone.0015473.s002.tif]

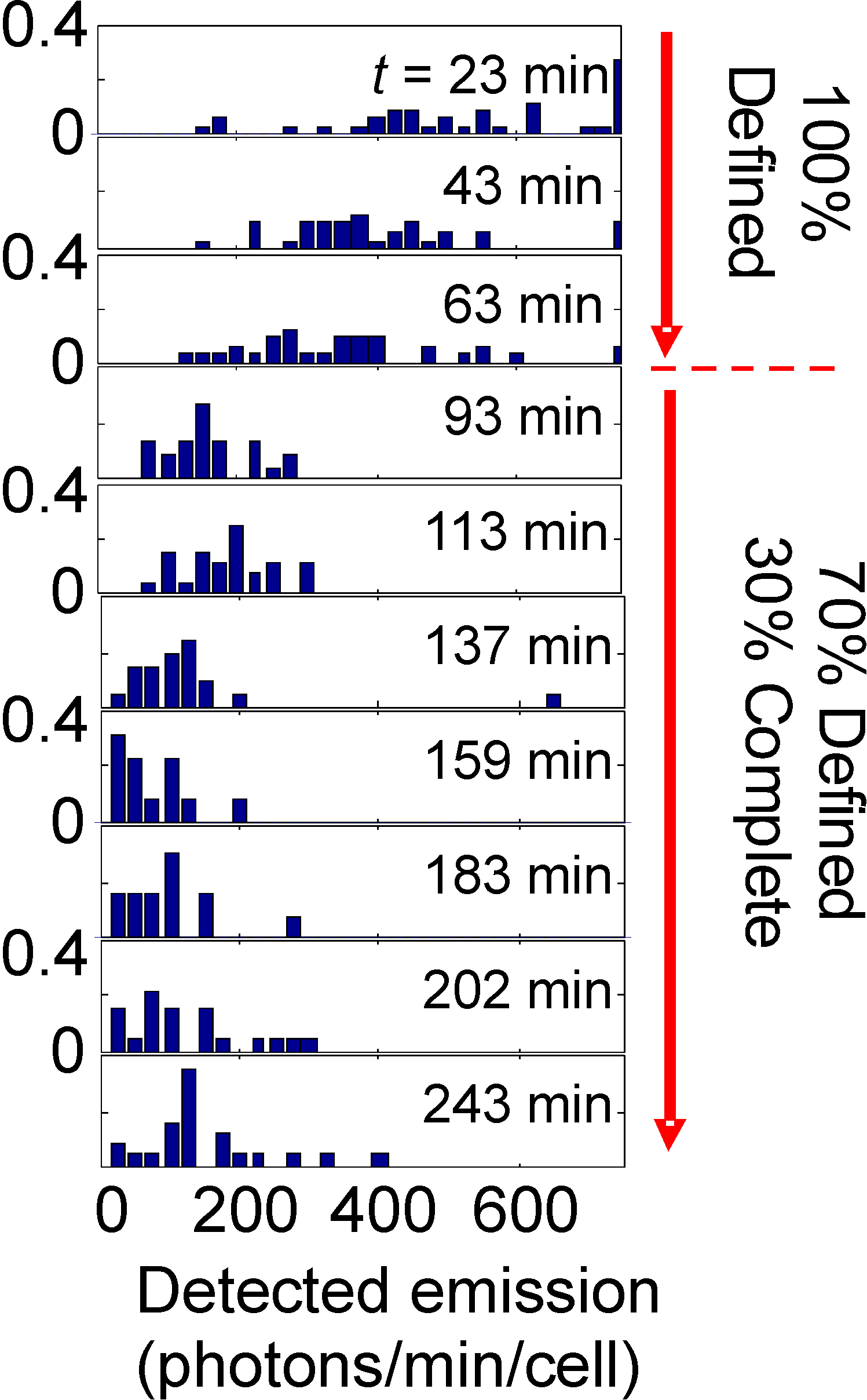

Supplement: Figure S3 — Inhibition of V.fischeri bioluminescence by complete (“rich”) medium. Light emission from individual cells in the perfusion chamber was tracked over time as the flowing medium was switched from an initial (100% defined medium) to a final (70% defined medium, 30% complete medium) composition. AI concentration remained 1000 nM at all times. Image times represent the starting time of a 16-minute bioluminescence exposure. The histograms, showing the fraction of observed cells emitting at the indicated level, collapse rapidly as complete medium is introduced. (TIF) [file pone.0015473.s003.tif]

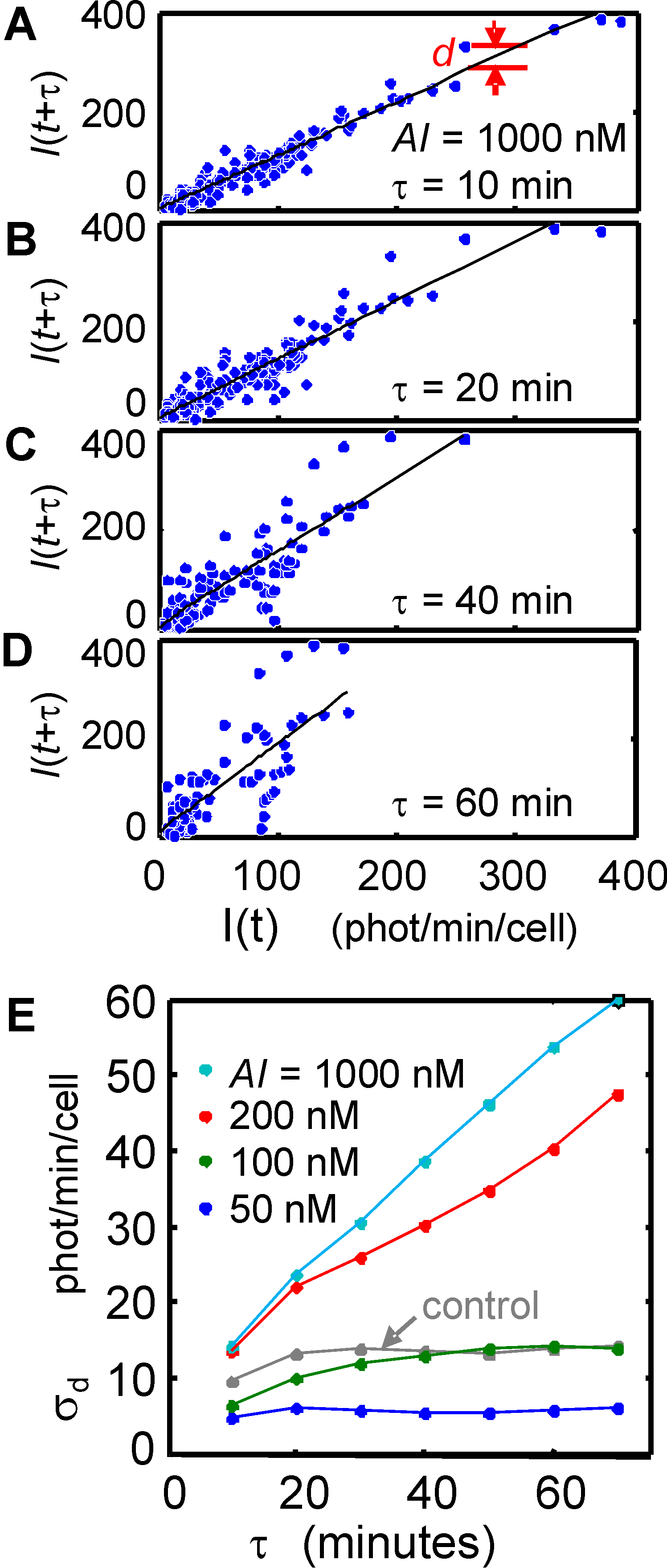

Supplement: Figure S4 — Temporal autocorrelation of individual cell luminescence. The emission level I(t) of a cell at time t is compared to its emission at a later time I(t+τ). Data represent individual cell emission levels measured at least 100 minutes after introduction of 1000 nM AI: (A)–(D) For small values of τ, the data are close to the (best fit) line, indicating that a cell's intensity at time t is a reasonably good predictor of its intensity at time t+τ. However as τ approaches 40–60 minutes, the scatter around the average line increases, indicating that the brightness of the cell at later times (relative to the average or best fit trend) is poorly predicted by its earlier brightness or by the average behavior of the other cells. The vertical distance d of each point from the trend line becomes larger at large τ. Panel (E) shows σd, (the standard deviation of d) as a function of τ. At high AI concentrations the standard deviation continues to grow for many minutes, indicating that the brightness of the cells continues to diverge both from its initial value and from the average growth trend. The σd of the control (fluorescence spheres) is essentially flat as expected, except for a dip near τ = 10 minutes (due to Gaussian filtering of the trajectories). (TIF) [file pone.0015473.s004.tif]
