# Supplementary material for: Heterogeneous Response to a Quorum-Sensing Signal in the Luminescence of Individual Vibrio fischeri
Source: PLoS One. 2010 Nov 16;5(11):e15473. doi: 10.1371/journal.pone.0015473 (PMC2982848; doi:10.1371/journal.pone.0015473)
Supplement: Text S1 — (DOC) [file pone.0015473.s005.doc]

**Text S1**

Heterogeneous response to a quorum-sensing signal in the luminescence of individual *Vibrio fischeri*

Pablo Delfino Pérez and Stephen J. Hagen

The bioluminescence of an individual bacterium generates an exceedingly weak optical signal. Measuring this signal quantitatively and over a period of several hours requires considerable care to ensure the stability of the optical configuration – *i.e.* to verify that there is minimal drift in the focus or movement in the cells adhered to the glass – and to verify that observed differences in light output from individual cells are statistically significant and not artifacts of the detection or image analysis. This supporting document provides further detail on these aspects. It also includes some additional experimental results and analysis, including brief summaries of additional control experiments that we performed .

**1. Physical stability**

We ensured the physical stability of the focus and the image scene (the cells in view) by collecting one dark field image (*i.e.* externally illuminated) between each pair of bioluminescence images for the duration of the observations – *i.e.* at ten minute intervals over the 4 hr duration of the observations. These dark field images provided a running check on the stability of individual cell position and focus. Owing to the thermal and mechanical stability of the microscope stage and optics (which were enclosed in a lightproof box on an optical table) small adjustments to the focus were only occasionally – and not always – needed after a few hours of observation.

**Figure S1** shows an excerpt from the image series collected for one cell. The dark field (gray scale) images show that the orientation and position of the cell are stable and are easily monitored. The image focus remains stable over time. However the emission level extracted from the luminescence (color scaled) images varies over time.

Cells did occasionally detach from the glass during measurement and were washed away by the flow. This was easily detected in the dark field images. Our data analysis (see *Materials and Methods*) addressed only those cells that remained in place during the entire observation period. In some experiments the microscope stage translated very slightly (few μm) during observations; this was trivially corrected in the image analysis and did not affect emission counts.

**2. Orientation of cells on glass**

Although the cells remain stationary during measurement, we cannot ensure that all cells lie completely flat on the glass coverslip. The orientation of the cell relative to the glass window cannot affect the direction of its light emission. It can however affect the image focus and therefore one needs to consider how this may affect the light collection efficiency. Cells that adhere to the glass window by standing upright (partially or completely) will give a less focused image than cells lying flat on the glass, and this will reduce the photon count per camera pixel. However we do not simply count the photons per camera pixel; we find the total count (above detector background) in a larger image region that surrounds a cell. (See *Materials and Methods.*) A partial separation of the cell body from the glass will slightly defocus the cell image, but it will not greatly affect the photon count in the larger image region. This should tend to reduce artifactual variations in cell brightness that owe to orientation of a cell relative to the glass surface. Moreover, for our data analysis we selected cells that appeared clearly focused, immobile, and well-demarcated through the entire series of dark field images. We did not attempt to track the emission from cells whose dark field images were inconstant or poorly resolved. It is not surprising that the majority of such stable, clearly-delineated cells were horizontal on the glass (see **Figure 2**), since the horizontal cells make maximum contact with the surface and are therefore in best focus and should adhere most strongly. The color scale of the bioluminescence image of **Figure 2** shows that, even among cells with similar size, shape, and orientation, the overall brightness varies noticeably. Therefore the specific orientation of the cells contributes very little to the observed variability in the detected cell luminescence.

**3. Noise levels**

It is important to verify that the observed cell-to-cell variations in emission are intrinsic to the luminescence and are not attributable to uncertainty in the detection. We established that the observed differences in the brightness of individual cells significantly exceed the uncertainty in detection.

The magnitude of the detection noise is apparent in the light levels measured from the fluorescent particles that we used as a control sample. See **Figure 3** and **Figure S2**. These particles generated a signal level (~10^2^ photons/minute/particle) comparable to *V.fischeri* but with a much smaller noise level of roughly 10-12 photons/minute/particle (rms) in 10 minute exposures collected over four hours. The brightness of the different control particles also showed a modest coefficient of variation (*cv* = standard deviation / mean) of 0.12. This is significantly narrower than the *cv* ≈ 1 seen in the brightness of individual *V.fischeri*.

The noise level can also be estimated from the magnitude of the parameter σ_d_(τ) which is shown in **Figure S4**. σ_d_(τ) describes the variance in the difference between the intensity *I*(*t*) of a cell and its intensity at a later time *I*(*t*+τ). (See *Autocorrelation of light output from individual cells*, below.) In the limit τ 🡪 0, σ_d_ extrapolates to roughly σ_d_ ~ 5-15 photons/minute/cell (**Figure S4**). This is consistent with the noise estimate from the control spheres.

Therefore the estimated noise level in the Gaussian-filtered (width σ = 10 minutes) emission *vs* time “trajectories” is of order 10 photons/minute. This is dominated by noise in the image intensifier as well as uncertainty in image background subtraction (see *Materials and Methods*). However it is much smaller than the observed variations (often ~100 photons/minute/cell) in the light output of different cells. Furthermore those large cell-to-cell variations are sustained over periods of hours in some cases and are qualitatively very dissimilar to the quiet signal obtained from the control spheres.

**4. Autocorrelation of light output from individual cells**

It is of interest to characterize the statistical properties of the luminescence behavior through its autocorrelation. However, the trend and divergence in the emission levels make this difficult: At high AI concentration, the cells not only become brighter over time but the brightness of each cell diverges further from the average brightness. **Figure S4** characterizes the autocorrelation behavior. The figure compares the brightness of each individual cell at time *t* to its brightness at a later time *t* + τ, as determined from all image frames collected at least 100 minutes after introduction of 1000 nM AI. The dashed line is the best fit line; it represents the average behavior of all cells under observation. The individual points are clustered near the best fit line (quite obviously in the plot for τ = 10 minutes). This is expected since the overall brightness of one cell rarely changes by large amounts over short time intervals; bright cells tend to remain bright and dark cells remain dark. However, for larger intervals τ the points become more widely scattered around the best fit line, indicating that as τ grows larger the brightness of a cell at time *t* becomes a progressively worse predictor of its brightness at *t* + τ. Although the average distance *d* of the points from the best fit line is zero, the standard deviation σ_d_ can be plotted as a function of τ. The bottom panel of the figure shows that σ_d_ saturates in ~30-40 minutes at a low AI concentration of 100 nM. (The cells remain fairly dark and therefore cannot diverge far from the average brightness.) However, at higher AI concentrations σ_d_ is still increasing at τ ~ 60 minutes. That is, upon the addition of 200-1000 nM AI, the brightness of each cell not only increases on average, but also diverges away from the population-averaged trend. This is a signature of the broad distribution of time scales that is present in the luminescent response of individual cells. (As expected, the control spheres show a constant σ_d_, except at τ < 20 minutes where the Gaussian filter smooths the noise.)

**5. Substrate deficiency**

We tested whether the variability in single-cell luminescence could be attributed to a shortage of the long chain aldehyde substrate that is needed for the bacterial luciferase reaction. The luciferase reaction involves the oxidation of tetradecanal (a C14 aldehyde) to produce the fatty acid (tetradecanoic acid), which is then recycled back to aldehyde [1, 2]. In cells that are deficient in the production of aldehyde, the addition of exogenous tetradecanal or tetradecanoic acid restores the C14 supply to the reaction cycle and stimulates a dramatic enhancement in luminescence output. We repeated our 1000 nM *AI* study in the presence of 1 μM tetradecanoic acid and found no difference in our results: over a four hour observation period, the average intensity of the cells, the distribution in onset times *t*_1/2_, and the variability in the light output were all similar to results observed in the absence of exogenous C14. The variability in luminescence output cannot be attributed simply to a deficiency of C14 substrate for the luminescence reaction.

**6. *QS* heterogeneity in a single colony**

Although we did not generally start each exponential culture from a single colony of MJ11 we did verify that the observed heterogeneity of bioluminescence emission was not due to a mixture of mutants. We grew an exponential (liquid) culture from an isolated single colony (selected from a photobacterium agar plate) and confirmed that in this culture both bright and dark cells were present at high *AI* concentrations (1000 nM *AI*). Cells originating from a single colony luminesced with different brightness levels under the same AI condition, even when nearby cells of equal size – lying just a few microns apart – were compared. This finding indicates that the heterogeneity cannot be easily attributed to appearance of a “dark mutant” in the strain. Anetzberger *et al.* report a similar result for *V.harveyi* luminescence [3].

**7. Rich medium effect**

Our observations confirm that individual cells exposed to new rich growth medium rapidly lose brightness, even with 1000 nM *AI* present. Luminescent cells transferred into fresh complete medium (even with exogenous *AI*) cease luminescence until they have grown for a period of time and (presumably) consumed or inactivated an inhibitor that is present in new medium [4, 5]. This “rich medium effect” was described in earlier literature on bulk cultures of *V.fischeri* although its cause has not been identified. Similar inhibition of homoserine-lactone dependent gene expression has also been observed in *Pseudomonas aeruginosa QS* [6] and has been attributed to a small molecule present in complete medium. In **Figure S3** luminescent MJ11 *V.fischeri* in defined medium and 1000 nM *AI* were switched at *t* ≈ 80 minutes to a mixture of defined medium (70%) and new complete medium (30%). Although the *AI* concentration was 1000 nM in both environments, the addition of complete medium causes the cell brightness histogram to collapse quickly to a lower average brightness. This response is much more rapid than the slower (~ hrs) timescale of response to added *AI.* For the luminescence studies described in the main text we avoided rich medium and used only defined medium.

References

1.     Ulitzur S, Hastings JW. (1978) Myristic acid stimulation of bacterial bioluminescence in aldehyde mutants. Proc Natl Acad Sci U S A 75(1): 266-269.

2.     Ulitzur S, Hastings JW. (1979) Evidence for tetradecanal as the natural aldehyde in bacterial bioluminescence. Proc Natl Acad Sci U S A 76(1): 265-267.

3.     Anetzberger C, Pirch T, Jung K. (2009) Heterogeneity in quorum sensing-regulated bioluminescence of *vibrio harveyi*. Mol Microbiol 73(2): 267-277.

4.     Kaplan HB, Greenberg EP. (1985) Diffusion of autoinducer is involved in regulation of the vibrio fischeri luminescence system. J Bacteriol 163(3): 1210-1214.

5.     Eberhard A. (1972) Inhibition and activation of bacterial luciferase synthesis. J Bacteriol 109(3): 1101-1105.

6.     Yarwood JM, Volper EM, Greenberg EP. (2005) Delays in pseudomonas aeruginosa quorum-controlled gene expression are conditional. Proc Natl Acad Sci U S A 102(25): 9008-9013.
